# Stress Echocardiography for Assessment of Diastolic Function

**DOI:** 10.1007/s11886-024-02142-2

**Published:** 2024-10-07

**Authors:** J. Lukas Laws, Tania Ruiz Maya, Deepak K. Gupta

**Affiliations:** Vanderbilt Translational and Clinical Cardiovascular Research Center, Division of Cardiovascular Medicine, Vanderbilt University Medical Center, 2525 West End Ave, Suite 300, Nashville, TN 37203 USA

**Keywords:** Echocardiography, Stress test, Diastolic dysfunction, Heart failure with preserved ejection fraction, Cardiomyopathy, Strain

## Abstract

**Purpose of Review:**

Diastolic dysfunction is an important, though often underappreciated, cause for exertional dyspnea. Echocardiography enables noninvasive evaluation of diastolic function and filling pressure, but images acquired at rest may be insensitive for detection of exertional abnormalities. This review focuses on stress echocardiography to assess diastolic function, including traditional and novel techniques, with emphasis on specific patient sub-groups in whom this testing may be valuable.

**Recent Findings:**

Emerging data informs patient selection for diastolic stress testing. Further, increasing literature provides considerations for performance and interpretation of diastolic metrics relevant to patients with heart failure with preserved ejection fraction, hypertrophic cardiomyopathy, athletes, and those with microvascular coronary dysfunction. Methods, such as speckle-tracking and multi-modality imaging, provide additional and complementary information for non-invasive diastolic assessment.

**Summary:**

This review serves as a guide to optimally utilize existing and novel techniques of stress echocardiography for diastolic assessment across a broad range of patients.

## Introduction

Exertional dyspnea is a common, but non-specific symptom, which may be due to cardiac causes. Although the dyspnea is exertional, the initial evaluation typically occurs at rest, e.g. laboratory, electrocardiogram, echocardiogram, and chest radiography. Consequently, under these non-exertional asymptomatic conditions, the electrocardiogram and echocardiogram may be unrevealing. The absence of overt resting cardiac dysfunction, e.g., reduced left ventricular (LV) ejection fraction, diastolic dysfunction, severe valvular disease, or pericardial disease, may belie a cardiac cause for exertional dyspnea. Therefore, a comprehensive evaluation for cardiac causes for exertional dyspnea requires interrogation of heart function under conditions which precipitate symptoms.

Diagnostic accuracy for cardiac etiologies for exertional symptoms is improved with provocative testing. This concept forms the basis for stress testing most commonly to evaluate for myocardial ischemia attributable to coronary artery disease. When a standard stress test is negative for myocardial ischemia, exertional dyspnea is often ascribed to a non-cardiac cause, but the cardiac evaluation should not stop. Indeed, assessment of cardiac structure and function and hemodynamic response with exertion is a key component of the investigation of unexplained exertional dyspnea as it may reveal exercise-induced: diastolic dysfunction, systemic or pulmonary hypertension, severe valvular regurgitation, chronotropic incompetence, or intracavitary gradients. This review focuses on the principles of stress echocardiography for assessment of diastolic function, with emphasis on conditions where this provocative testing may be of particular diagnostic value.

## Assessment of Diastolic Parameters on Echocardiography

Echocardiography can non-invasively estimate cardiac filling pressures and flow yielding similar quantitative parameters to those obtained from invasive hemodynamic assessment [1, 2]. Various resting echocardiographic parameters can be used to estimate left sided filling pressure as LV end-diastolic pressure, mean left atrial (LA) pressure, or mean pulmonary capillary wedge pressure (PCWP). Only LV diastolic pressure is elevated in early-stage diastolic dysfunction, with normal left atrial and pulmonary pressure. The basis of diastolic stress testing is transduction of this elevated LV diastolic pressure with tachycardia or increased LV afterload to the left atrial pressure as measured by PCWP. This can be evaluated on stress echocardiography by measurement of mitral E/e’ ratio to estimate PCWP and estimating pulmonary artery systolic pressure (PASP) using the velocity of regurgitant blood through the tricuspid valve (TR Vmax). Measurement of resting and exercise mitral inflow velocity (E), septal and lateral tissue mitral annular tissue velocity (e’), and TR Vmax should be part of the routine assessment of diastolic function at rest and obtained in a protocolized manner during stress echocardiography.

Peak mitral inflow to mitral annular velocity ratio (E/e’) is the most studied parameter in relation to diagnosis and prognosis with diastolic stress testing. E/e’ is calculated from the ratio of peak velocity of antegrade blood flow through the mitral valve using pulse wave spectral Doppler (E) at the leaflet tips to the LV regional lengthening velocity at the mitral annulus (e’) during early diastole. Healthy myocardium will have proportional increases in transmitral flow and myocardial relaxation velocities during exercise, such that the E/e’ ratio remains within a normal range. Diseased myocardium exhibits diastolic dysfunction ranging from impaired relaxation to increasing non-compliance. As left atrial pressure rises, this is reflected as higher peak E velocity, and as ventricular diastolic dysfunction worsens, mitral annular e’ velocities decline. This combination manifests on echocardiography as higher E/e’ ratio in diseased myocardium [3–5]. The E/e’ ratio has been validated against invasive hemodynamic assessment of PCWP during exercise, the clinical standard for diagnosis of diastolic dysfunction, as well as diminished exercise capacity on cardiopulmonary exercise test (CPET). Tissue velocity can be measured at the septal and lateral mitral annulus, with lateral wall e’ velocities generally higher. A cut-off value of average E/e’ > 14 is suggested as the threshold for a diagnosis of diastolic dysfunction, with optimal specificity of 86% but limited sensitivity for the diagnosis (46%) compared with CPET [6]. As cardiac pathology such as regional wall motion abnormalities from ischemic heart disease or valvular calcification may render one of these values inaccurate, specific thresholds of septal E/e’ > 15 or lateral E/e’ > 13 can also be used as a surrogate for elevated PCWP [1]. Comparison of the change in average E/e’ from each patient’s individual baseline is also important, as a proportional E/e’ increase more than 1.5 times the resting value is also suggestive of diastolic dysfunction [7].

Stress echocardiography allows for and should include assessment of other parameters which enhance evaluation of diastolic function. Left atrial volume index is an important parameter for resting assessment of diastolic function, as chronically elevated LA filling pressures induce eccentric atrial remodeling. But data for LA volume changes during exercise are less defined, and LA volume measurement is not a routine measurement during stress echocardiography. Right ventricular systolic pressure (RVSP) is a non-invasive tool to estimate the pulmonary artery systolic pressure (PASP) paralleling measurement of these by invasive catheterization. Using continuous wave spectral Doppler through the tricuspid valve during stress, a peak tricuspid regurgitant (TR) velocity > 3.4 m/s provides incremental specificity to identify diastolic dysfunction [8]. In contrast to E/e’ ratios, increase in PASP is an expected finding during exercise, with athletes capable of generating higher PASP compared to sedentary controls. Exercise PASP must be interpreted in context of the patient’s resting TR velocity, as well as their expected aerobic capacity for age [9, 10]. Thus PASP is an important component of the diastolic stress test and should be interpreted in concert with E/e’ rather than in isolation. Mounting evidence also supports PASP evaluation in relation to augmentation of cardiac output (CO) with exercise, as those with left heart dysfunction have significantly increased PASP/CO slope compared to athletes and healthy controls [11].

## Patient Selection and Stress Test Protocol

**Fig. 1:**
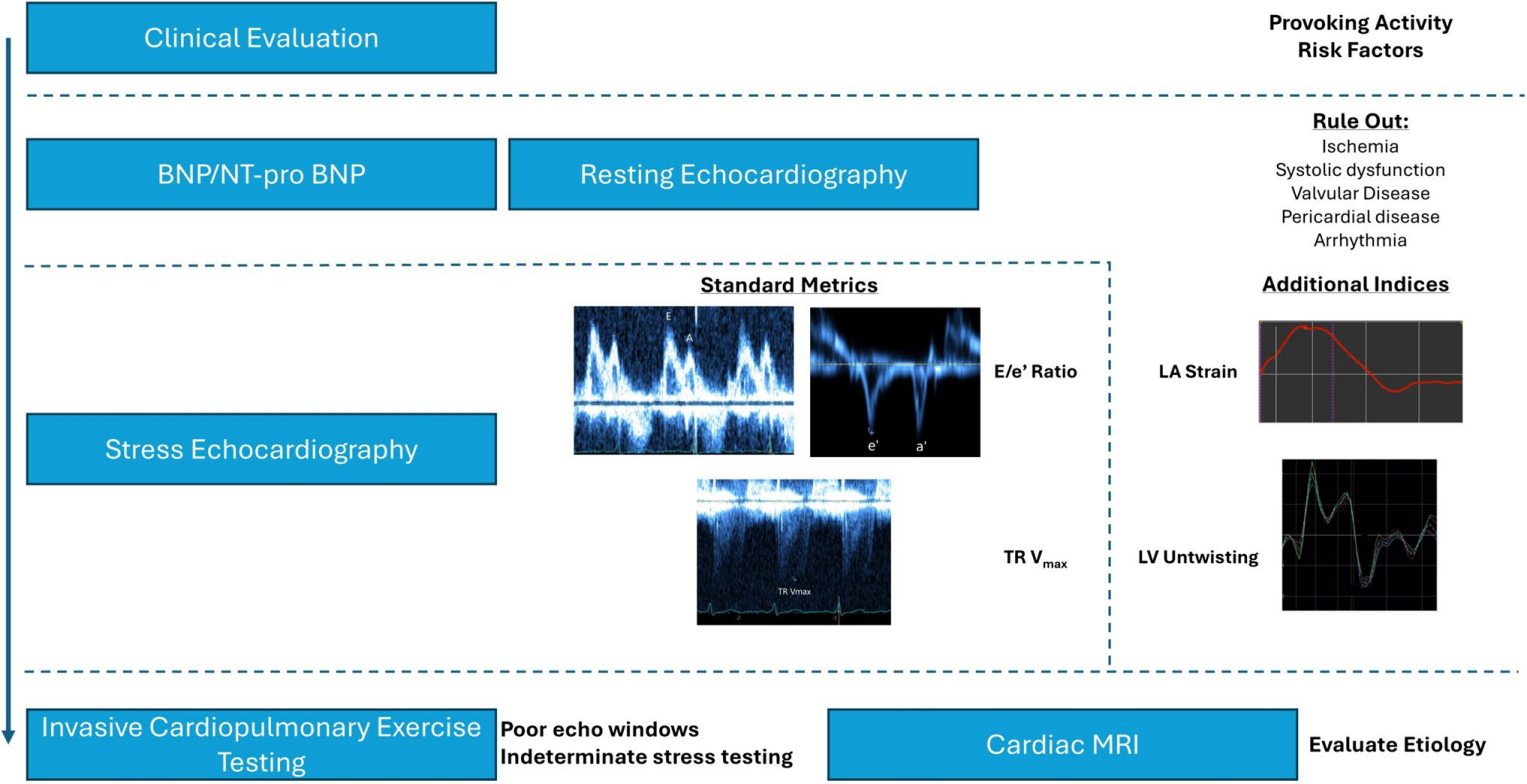
Stepwise approach to assessment of diastolic function. Stress echocardiography is a critical step in the diagnostic algorithm to evaluate diastolic dysfunction as a cause of exertional dyspnea. Beginning with clinical evaluation and resting echocardiography, stress echocardiography using E/e’ and TR Vmax along with additional resting indices of speckle-tracking LA strain and LV apical untwisting can help clinch the diagnosis for those with intermediate pre-test probability. If needed, invasive evaluation in select populations and cardiac MRI for evaluation of etiology are also key for completing the evaluation. NT-pro BNP = N-terminal pro B-type natriuretic peptide. TR = tricuspid regurgitation. LA = left atrium. LV = left ventricle. MRI = magnetic resonance imaging

Diastolic stress testing is indicated for the evaluation of exertional dyspnea when the etiology of symptoms remains unresolved. Clinical suspicion for exertional dyspnea attributable to diastolic dysfunction should be raised by the presence of risk factors for diastolic dysfunction or borderline values from previous testing detailed in Table 1. For example, some guideline statements recommend N-terminal pro B-type natriuretic peptide (NT-pro BNP) values greater than 220 pg/ml or BNP values greater than 80 pg/ml should prompt consideration of heart failure as a cause of dyspnea [8]. It is important, however, to consider biomarker evaluation as a spectrum rather than a dichotomous decision to pursue further testing. While NT-pro BNP < 125 pg/ml and BNP < 35 pg/ml are strong predictors of a noncardiac cause of dyspnea, NT-pro BNP values within the “normal range” were falsely low in 18% of patients with invasive hemodynamic confirmed Heart Failure with Preserved Ejection Fraction (HFpEF) [12, 13]. Similarly, higher resting E/e’ and left atrial volume index (LAVi) values, e.g., > 8 and 28 ml/m2, respectively, that approach but do not yet surpass threshold criteria for diastolic dysfunction should raise suspicion for exertional diastolic dysfunction, particularly in the absence of systolic dysfunction [14]. Clinically validated decision aids, such as the HF2PEF or HFA-PEFF score, may help select patients for further testing to evaluate for cardiac causes of dyspnea [8, 15]. A stepwise approach to diastolic stress testing using these metrics is summarized in Fig. 1.

**Table 1:** Patient selection for diastolic stress testing

Symptoms	Risk Factors	Lab Values	Resting Echocardiography	Cardiac MRI
Unexplained exertional dyspnea	Diabetes Mellitus Hypertension Obesity Physical Inactivity Female Sex Renal dysfunction Atrial fibrillation	NT-pro BNP > 125 pg/ml BNP > 35 pg/ml	E/e’ > 8 LA volume index > 28 ml/m2 LA reservoir strain < 25% LV GLS <|16%|	LA volume index > 52 ml/m2

For patients with exertional dyspnea, diastolic stress testing can help identify a cardiac etiology. Risk factors and findings from laboratory and resting imaging define high pretest probability of diastolic dysfunction. *NT-pro BNP *N-terminal pro B-type natriuretic peptide, *LA * left atrium, *LV*  left ventricle, *GLS *global longitudinal strain, *MRI*  magnetic resonance imaging

The choice of stress modality is important for optimal data collection and image acquisition. In the United States, treadmill testing using the Bruce protocol is commonly utilized, typically for wall motion assessment to evaluate for ischemia [16]. Supine bicycle protocols, used more commonly in Europe, are optimal for diastolic stress testing as a power output can be more specifically defined to better assess diastolic reserve [7]. While diastolic abnormalities may persist briefly after exercise, optimal assessment is obtained during exercise as the supine bicycle facilitates apical window acquisition. Images should be captured at low-level and sub-maximal exercise, with attention to circumstances that lead to reproduction of symptoms that prompted clinical evaluation. Often at low levels of tachycardia (heart rate [HR] > 110 bpm) fusion of the E and A waves of mitral inflow occurs such that the sub-maximal exercise imaging should be targeted around this HR interval [6, 17].

For patients unable to exercise, pharmacologic stress testing currently has little role for diastolic assessment, as agents such as dobutamine, not only enhance chronotropy and inotropy, but also augment lusitropy, which may obscure underlying diastolic dysfunction. Isometric hand grip may be an alternative for those unable to perform the supine bicycle or walk on the treadmill [18]. Limited studies evaluated the role of volume load during noninvasive testing, achieved via passive leg raise or saline bolus [19, 20]. When exercise as a stress modality is not feasible, these options are reasonable alternatives for noninvasive assessment, as invasive CPET relies on volume challenge to test diastolic response for physically-limited patients.

## Beyond Traditional Markers of Diastolic Dysfunction

Stress echocardiography is an excellent non-invasive method to identify diastolic dysfunction across a broad cohort of patients, but interpretation of the findings can be augmented by using speckle-tracking technology and a multimodality approach. LV apical rotation, left atrial strain, and cardiac magnetic resonance imaging (MRI) are tools increasingly used by the cardiologist for assessment of diastolic dysfunction and can add incremental value for diagnosis and prognosis.

### LV Apical Rotation

Emerging parameters also show promise for detection of early-stage diastolic dysfunction. Early diastolic pathology is manifest as loss of suction of the LV cavity that normally facilitates transit of blood once the mitral valve opens during early diastole. LV suction is of particular importance in exercise as the diastolic time is shortened. Speckle-tracking technology can be applied to parasternal short axis views of the apex to measure LV untwisting velocity and torsion to quantify LV suction [21]. These values of circumferential strain and strain velocity give an estimate of LV untwisting function that has been validated against invasive measurement of LVEDP [22]. Reduced LV untwisting at rest has been shown to be predictive of exercise-induced diastolic dysfunction when other resting parameters are nondiagnostic, and correlates well with LV filling pressure during exercise [23, 24].

### Left Atrial Strain

Echocardiographic assessment of LV diastolic dysfunction with E/e’ and TR Vmax are reliant on the typical cascade of pathophysiology of LV diastolic dysfunction: decreased LV suction progresses to left atrial hypertension and remodeling, leading to pulmonary hypertension. E/e’ focuses on assessment of LV relaxation in relation to passive and active atrial filling, while TR Vmax focuses on transduction of elevated filling pressure to the pulmonary vasculature. Dedicated assessment of the left atrium and its contribution to diastolic function is not comprehensively assessed with these traditional methodologies. Left atrial speckle-tracking strain analysis improves left atrial assessment by evaluation of atrial function throughout the cardiac cycle. Apical 4-chamber LA images are typically used to estimate global LA strain parameters, and full segmental strain may be evaluated by concurrent speckle-tracking of the apical 2-chamber as well as short axis images of the left atrium [25]. Left atrial function is evaluated in three distinct phases: reservoir, conduit, and booster. Reservoir function focuses on atrial filling during ventricular systole, a measure of compliance but also of effective emptying. Conduit function evaluates the change in atrial wall motion during passive diastolic ventricular filling. Booster (or contractile) strain evaluates the robustness of atrial contraction at the end of ventricular diastole. Strain imaging has advantages over E/e’ evaluation as it is not angle-dependent and independent of cardiac translational motion, and has been shown to closely correlate with LV end diastolic pressure [26]. LA strain has been best validated against invasive hemodynamics using resting images, but there may be promise for applying this modality during stress as well, specifically for the diagnosis of HFpEF discussed below. Speckle-tracking strain is universal across imaging modalities, and can be used across echocardiography, cardiac computed tomography, and cardiac MRI [27]; although there is variation in normative values by modality and for each vendor [28].

### Cardiac Magnetic Resonance Imaging

Use of cardiac MRI (CMR) for the diagnosis of cardiac pathology is growing, and CMR correlates of diastolic dysfunction are of increasing importance. Beyond assessment of diastolic function, CMR plays a critical role in establishing the etiology of diastolic dysfunction. LV mass and LA volume are better assessed on CMR compared to echocardiography, and provide better prediction of LV filling pressures and association with cardiovascular mortality [29]. Resting CMR for tissue characterization provides complementary information to stress echocardiography, but real-time CMR with exercise has also been investigated as a modality to diagnose HFpEF, with LA long axis strain the best predictor [30]. While most patients with HFpEF have systemic microvascular and metabolic dysfunction, recognition of diastolic dysfunction may be the first manifestation of infiltrative disease, genetic cardiomyopathies, or epicardial ischemic disease that can be accurately evaluated with noninvasive tissue assessment on CMR. Stress echocardiography will remain the gold standard for real-time assessment of exercise hemodynamics, but coupled with CMR imaging becomes a potent diagnostic combination across the spectrum of diastolic pathology.

## Diagnostic Utility and Management Implications for Specific Populations

### Heart Failure with Preserved Ejection Fraction

The most common diagnosis achieved through diastolic stress testing is Heart Failure with Preserved Ejection Fraction. Diastolic dysfunction is responsible for exertional symptoms in up to 40% of patients presenting with heart failure, so should be strongly considered when a patient presents with other findings suggestive of heart failure [31]. Even when resting echocardiography and biomarker evaluation is inconclusive, about 30% of HFpEF patients with normal resting filling pressures will have elevated filling pressures with exercise that can be diagnosed with stress echocardiography [32]. Stress echocardiography is most accurate for diagnosing HFpEF using E/e’ > 15 alone, but evaluation of exercise-induced pulmonary hypertension with TR Vmax > 3.4 m/s improves diagnostic sensitivity [8]. In one study evaluating the use of diastolic stress testing in patients with HFpEF, E/e’ > 15 was only 45% sensitive for the diagnosis compared to invasive CPET. There was a strong association of E/e’ with CPET-derived peak VO2, even at E/e’ levels below 15 [33]. HFpEF does not emerge suddenly, progressive loss of diastolic reserve hits a critical level to produce symptoms, and that level is likely variable across individuals. In this patient population, it is important to consider disease on a spectrum rather than at a diagnostic threshold.

The left atrium plays an important role in the cascade of elevated filling pressure in HFpEF patients to the pulmonary and right sided circulation, and resting strain evaluation has been associated with outcomes in HFpEF [34, 35]. LA strain assessment is not currently widely adopted as a routine part of stress echo diastolic evaluation of patients with HFpEF, but some emerging evidence suggests promise as an augmentation to traditional parameters. A recent study demonstrated exercise LA conduit strain as having good predictive capability of invasive exercise-induced PCWP elevation [36]. In the HFpEF-Stress trial, however, resting LA compliance was a better indicator of prognosis than exercise LA compliance [37]. Rest and exercise strain were shown to provide additional insight into atrial myopathy in a HFpEF population when coupled with exercise E/e’ and invasive filling pressure measurement [38]. Evaluation of exercise-induced changes in left atrial filling pressure using exercise strain may also help select patients most likely to benefit from developing technologies for atrial shunting in HFpEF [39].

### Hypertrophic Cardiomyopathy

Exercise stress echocardiography is a standard test for assessment of for patients with symptomatic hypertrophic cardiomyopathy (HCM) [40]. The focus of the test is on provokable left ventricular outflow tract obstruction, but assessment of diastolic function can help identify the causative factors of dyspnea in HCM patients and facilitate targeted management of that pathology. In a study evaluating concurrent stress echocardiography with CPET, diastolic dysfunction assessed by E/e’ and PASP was the main driver of exercise limitation in HCM patients, and was associated with development of ultrasonographic evidence of pulmonary edema [41, 42]. This may be an even more important component of the evaluation in patients with apical HCM where LV outflow obstruction or poor exertional stroke volume augmentation are less likely to contribute to symptoms. In this subset of patients, adverse LV remodeling from diastolic dysfunction is associated with poor functional status [43]. As dynamic changes in heart rate are inevitably linked with changes to cardiac contraction and relaxation, there is interest in exploring systolic-diastolic coupling in HCM to better assess the interplay of these factors [44]. Now that targeted therapies for HCM are available with cardiac myosin inhibitors, diastolic assessment on serial stress echocardiography may be another therapeutic target, as secondary analysis of the EXPLORER-HCM trial demonstrated that treatment with a cardiac myosin inhibitor was associated with improved metrics of diastolic function and improved left atrial remodeling [45].

### Athletic Left Ventricular Remodeling

The initial evaluation of progressive dyspnea in athletes is a unique challenge due to supranormal exercise capacity compared with healthy non-athlete controls, although it will be below their own expectations or insufficient to meet the needs of competitive demands. Physiologic adaptation to exercise can often mimic pathologic remodeling in early stages and obscure diagnosis from resting images [46]. Aerobic training is a potent stimulator of concentric remodeling, but also a potent driver of calcium channel handling that preserves, and augments, diastolic reserve capacity. E, A, and e’ velocities are all higher in athletes compared to healthy controls, resulting in a low E/e’ ratio [47, 48]. The TR Vmax is also not an independent predictor of diastolic function in athletes as a rise in pulmonary pressure is a normal response to exercise in trained aerobic athletes [47]. Assessment of diastolic parameters is a crucial part of stress echocardiography evaluation in athletes to rule out phenocopies of athletic remodeling such as HCM. This is of particular importance because aerobic remodeling can still result in intracavitary gradients and systolic anterior motion of the mitral valve leading to an inappropriate diagnosis of HCM that limits athletic participation [49]. Using the typical cutoff value of average E/e’ > 14 with exercise is highly specific for distinguishing athletic remodeling from HCM; however, lacks sensitivity, particularly when the requirement to distinguish these subgroups arises in a clinical context of high baseline cardiovascular fitness. In a study by Finocchairo et al., the optimal cutoffs on resting imaging to differentiate physiology athletic remodeling from diastolic dysfunction in HCM were septal E’ < 10 cm/s and lateral E’ < 12 cm/s [50].

### Microvascular Coronary Disease

Evaluation for epicardial coronary disease is an important part of the workup for exertional dyspnea, but a lack of macrovascular coronary stenosis does not absolve the coronary circulation of contribution to symptomology. Microvascular coronary disease, driven by oxygen supply/demand mismatch and deranged nitric oxide signaling can produce similar symptoms and is an important component of HFpEF pathophysiology [51, 52]. A comprehensive stress echocardiographic evaluation can further define the mechanistic contribution to symptoms by evaluating the coronary flow reserve (CFR) under vasodilator stress. In the PROMIS-HFpEF study, HFpEF patients with CFR < 2.5 had impaired subendocardial systolic and diastolic function, defined by LV global longitudinal strain and LA reservoir strain, respectively. These metrics were predictive of impaired functional status in those with microvascular dysfunction beyond E/e’ values in isolation [53]. Microvascular angina evaluation is demonstrative of the multimodality approach needed to identify and direct appropriate therapy for those with exertional dyspnea.

## Validation with Invasive Testing

Despite advances in stress echocardiography, invasive CPET remains the gold standard for excluding diastolic dysfunction as the cause of dyspnea. Therefore, diastolic parameters during stress echocardiography were validated against invasive hemodynamic assessment of PCWP, mean LVDP, or change in LV minimal pressure [14, 23, 54, 55]. The mode of exercise in all these studies was supine bicycle. In one prospective study evaluating twelve patients with exertional dyspnea, simultaneous echocardiography and cardiac catheterization during exercise demonstrated an E/e’ > 15 during exercise associated with a significantly elevated PCWP > 20 mm Hg. The sensitivity of an E/e’ < 15 as a predictor for normal PCWP was 89% [55]. A more recent prospective study of 74 patients (50 with HFpEF, 24 with noncardiac dyspnea) who underwent simultaneous echocardiography and catheterization at 20W exercise demonstrated only a modest correlation between E/e’ medial vs PCWP (*r* = 0.57, *p* < 0.0001) and E/e’ lateral vs PCWP (*r* = 0.54, *p* < 0.0001). The authors noted that the relationship between E/e’ and PCWP changed from rest to exercise, with PCWP values that were higher for a given E/e’ with exercise compared to rest [14].

## Limitations of Non-Invasive Testing

Diastolic stress echocardiography is most useful in patients with intermediate pretest probability, due to the high specificity of exercise E/e’ offering strong evidence for a diagnosis of HFpEF. In contrast, diastolic stress testing has lesser utility in patients with either a very low or very high pretest probability, in which case making a clinical diagnosis often obviates the need for additional testing. Once a patient is diagnosed with exercise-induced diastolic dysfunction, the focus of additional testing should be directed towards identification and treating the underlying cause, e.g., uncontrolled hypertension, infiltrative cardiomyopathy, or diabetic cardiomyopathy.

Furthermore, E/e’ may be inaccurate in patients with mitral annular calcification, moderate to severe mitral regurgitation, constrictive pericarditis, mitral valve replacement or repair, left bundle branch block, or significant aortic regurgitation. Similarly, estimating PASP may be challenging if tricuspid regurgitation is absent or may be underestimated in cases of severe tricuspid regurgitation or right ventricular dysfunction. In patients with the previously mentioned pathologies, it is reasonable to forego diastolic stress echocardiography and proceed with invasive testing. Patients with inadequate or equivocal resting echocardiogram images may also be considered for direct invasive testing, due to the high likelihood that their diastolic stress echocardiogram will also be equivocal. In the cardiac catheterization lab, hemodynamics should be obtained at rest and with exercise.

## Future Directions

New diastolic stress echocardiographic parameters continue to be assessed, including the ratio of early diastolic mitral inflow velocity to flow propagation velocity (E/Vp), diastolic functional reserve index (DFRI) based on changes in e’ velocity during exercise, isovolumic relaxation time (IVRT), LV diastolic strain rate, and diastolic dyssynchrony [56]. However, these markers still require validation and thus, E/e’ and PASP remain the most reliable echocardiographic indicators of LV filling pressure. The addition of machine learning algorithms and artificial intelligence also has promising implications for the future of this modality [57].

## Conclusion

Stress echocardiography for assessment of diastolic function is now a well-established, albeit underutilized, tool for the evaluation of cardiac dyspnea. It is widely available, reproducible, and applicable to a diverse cohort of cardiac patients. By incorporating stress echocardiography with a multimodality imaging approach, cardiologists can optimally diagnose diastolic dysfunction and provide targeted therapy.

## Key References

- Omote K, Sorimachi H, Obokata M, Reddy YNV, Verbrugge FH, Omar M, et al. Pulmonary vascular disease in pulmonary hypertension due to left heart disease: pathophysiologic implications. Eur Heart J. 2022;43:3417–31.
  - Evaluation of the pulmonary circulation during exercise with stress echocardiography is an important component that can help further elucidate the etiology of exertional dyspnea that may aid with treatment decisions.
- Backhaus SJ, Schulz A, Lange T, Schmidt-Schweda LS, Hellenkamp K, Evertz R, et al. Prognostic and diagnostic implications of impaired rest and exercise-stress left atrial compliance in heart failure with preserved ejection fraction: Insights from the HFpEF stress trial. Int J Cardiol. 2024;404:131949.
  - This study demonstrates the utility of atrial strain at rest to identify diastolic dysfunction and those at risk of poor outcomes.
- Litwin SE, Komtebedde J, Hu M, Burkhoff D, Hasenfuß G, Borlaug BA, et al. Exercise-Induced Left Atrial Hypertension in Heart Failure With Preserved Ejection Fraction. JACC Heart Fail. 2023;11:1103–17.
  - Stress echocardiography has increasing implications for clinical management, as highlighted by this study that identifies patients most likely to benefit from novel atrial shunting therapy.

## Data Availability

No datasets were generated or analysed during the current study.
